# Correction: A scoping review protocol on childhood immunization reminder strategies available to parents in Canada and the United States of America

**DOI:** 10.1371/journal.pone.0347238

**Published:** 2026-04-14

**Authors:** Matilda Anim-Larbi, Vivian Puplampu, Sithokozile Maposa, Akram Mahani, Mary Chipanshi

The first author, Matilda Anim-Larbi, is incorrectly noted as the corresponding author. The correct corresponding author is Sithokozile Maposa. Sithokozile Maposa’s email address is: s.maposa@usask.ca.
