# Supplementary material for: A scoping review protocol on childhood immunization reminder strategies available to parents in Canada and the United States of America
Source: PLoS One. 2025 May 22;20(5):e0323186. doi: 10.1371/journal.pone.0323186 (PMC12097596; doi:10.1371/journal.pone.0323186)
Supplement: S1A Table — (DOCX) [file pone.0323186.s001.docx]

**S1A Table: Literature search terms using CINAHL**

| Search ID | Search Terms | Search Options | Last Run Via |
| --- | --- | --- | --- |
| S7 | S5 AND S6 | Expanders-Apply equivalent search  Find all search terms | EBSCOhost research databases  Advanced search  CINAHL Plus with full text |
| S6 | (MH “Canada+”) OR (MH “United States+”) | Limiters-Publication date 2015/01/01-2025 | EBSCOhost research databases  Advanced search  CINAHL Plus with full text |
| S5 | S1 AND S2 AND S3 | Limiters-Publication date  2015/01/01-2025 | EBSCOhost research databases  Advanced search  CINAHL Plus with full text |
| S4 | S1 AND S2 AND S3 | Expanders-Apply equivalent search  Find all search terms  Limiters-all child ie., infants, preschool, newborn | EBSCOhost research databases  Advanced search  CINAHL Plus with full text |
| S3 | reminder* or prompt* or "text* message* reminder*" or text* or email* or call* or "reminder system*" or "digital reminder*" or phone call* or "telephone call*" or "follow up call*" or follow-up or following-up. | Expanders-Apply equivalent search  Find all search terms | EBSCOhost research databases  Advanced search  CINAHL Plus with full text |
| S2 | vaccin* or immuni* or inoculat* or (MH Immunization) | Expanders-Apply equivalent search  Find all search terms | EBSCOhost research databases  Advanced search  CINAHL Plus with full text |
| S1 | parent* or caregiver* or mother* or father* or guardian* or famil* or (MH Parent) | Expanders-Apply equivalent search  Find all search terms | EBSCOhost research databases  Advanced search  CINAHL Plus with full text |
