# Supplementary material for: A scoping review protocol on childhood immunization reminder strategies available to parents in Canada and the United States of America
Source: PLoS One. 2025 May 22;20(5):e0323186. doi: 10.1371/journal.pone.0323186 (PMC12097596; doi:10.1371/journal.pone.0323186)
Supplement: S1B Table — (DOCX) [file pone.0323186.s002.docx]

**S1B Table. Three-step literature search strategy.**

| Step 1 | Step 2 | Step 3 |
| --- | --- | --- |
| Searching of relevant articles from CINAHL database. | The second search will involve three databases: CINAHL, Medline (Ovid), Embase (Elsevier) | Additional strategies that include hand-searching of sources such as google scholar, and reference list of selected articles.  Full-text articles of gray literature will be selected from internet sources such as google scholar  Pertinent references  Expert opinion will be sought to identify relevant data. |
