# Supplementary figures and images for: A scoping review protocol on childhood immunization reminder strategies available to parents in Canada and the United States of America
Source: PLoS One. 2025 May 22;20(5):e0323186. doi: 10.1371/journal.pone.0323186 (PMC12097596; doi:10.1371/journal.pone.0323186)

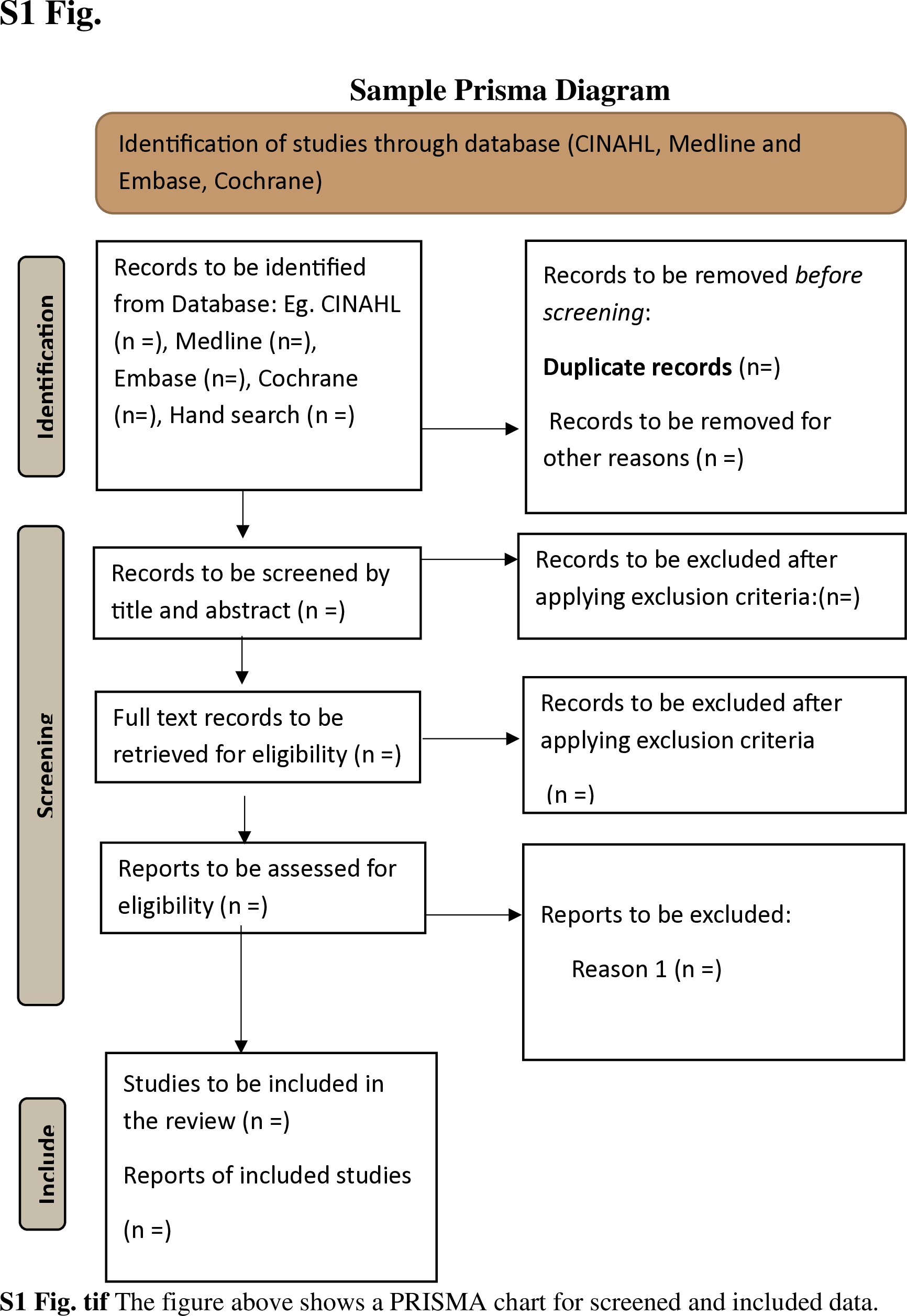

Supplement: S1 Fig — (TIF) [file pone.0323186.s003.tif]
