# Supplementary material for: A scoping review protocol on childhood immunization reminder strategies available to parents in Canada and the United States of America
Source: PLoS One. 2025 May 22;20(5):e0323186. doi: 10.1371/journal.pone.0323186 (PMC12097596; doi:10.1371/journal.pone.0323186)
Supplement: S1C Table — (DOCX) [file pone.0323186.s004.docx]

**S1C Table. Scoping review data extraction stages.**

| No | Stage | Task | Reason/s |
| --- | --- | --- | --- |
| 1. | Stage 1 | Title and abstract screening | To ensure that the articles are eligible for full text screening |
| 2. | Stage 2 | Full text screening | Information about vaccine reminders will be found in the methods and results section |
| 3. | Final stage | To resolve conflicts | To ensure that articles that do not fully meet eligibility and the two independent reviewers do not agree to their inclusion, a third reviewer will be invited to resolve the conflicts. |
