# Supplementary material for: A scoping review protocol on childhood immunization reminder strategies available to parents in Canada and the United States of America
Source: PLoS One. 2025 May 22;20(5):e0323186. doi: 10.1371/journal.pone.0323186 (PMC12097596; doi:10.1371/journal.pone.0323186)
Supplement: S1D Table — (DOCX) [file pone.0323186.s005.docx]

**S1D Table. Scoping review protocol data extraction table**

| Author/date | Thesis statement/Purpose | Method | Findings/Themes | Relevance to the topic |
| --- | --- | --- | --- | --- |
|  |  |  |  |  |
