# Supplementary material for: A scoping review protocol on childhood immunization reminder strategies available to parents in Canada and the United States of America
Source: PLoS One. 2025 May 22;20(5):e0323186. doi: 10.1371/journal.pone.0323186 (PMC12097596; doi:10.1371/journal.pone.0323186)
Supplement: Attachment — Submitted filename: Revision Table Feb 7.docx [file pone.0323186.s008.docx]

**A Scoping Review Protocol on Childhood Immunization Reminder Strategies Available to Parents in Canada and the United States of America - Revisions**

| No. | Reviewers’ comments | Revisions |
| --- | --- | --- |
|  | **Reviewer #1**: This paper describes a scoping review to map the literature on immunization reminder strategies for parents. As we are in a time of declining immunizations, this work is very important and could potentially be very impactful. However, there is a lack of clarity around the specific rationale/focus for this study, and it is unclear why the authors are embarking on a scoping review and not a systematic review for this work, particularly as many of the methods pertain to a systematic review protocol | Thank you for the comment. We have revised the manuscript to enhance clarity and focus of the review which is on immunization reminder strategies available to parents in North America focusing on Canada and the United States.  We concur with you that our steps we took in the scoping revie methodology was thorough and the methodology could be revised to systematic review. The first author, who is a PhD student contributed about 85% percent of the time to the review and since there were not two independent reviewers for the review we decided to keep the review as a scoping review. More importantly is our focus for conducting the review which was to scope the literature and map out the size of published work on the topic which fit well with the reason for conducting a scoping review thus we would like to keep the work as a scoping review. We could consider a revised title as “Systematized Review” to align with Grant and Booth (2009) classification for review conducted by a graduate student. |
|  | **Abstract**  **Reviewer #1**  The abstract includes the phrase, “with a focus on supporting Black parents”, but what does this specifically mean? Does this mean immunization reminders are needed for Black parents in particular due to vaccination rates, or is it that reminders have been found to be particularly useful to this group of parents? Additional information in the background/rationale of the paper to justify this focus would be helpful | Thank you for the comments.  We concur with the reviewer on the need to clarify the statement “with a focus on supporting Black parents”, vaccine reminders will be beneficial to both the parents and Black population.  The statement below is now added in the abstract.  Immunization reminders can help parents who forget or miss vaccination appointments. In the USA, current literature indicates that Black children have lower vaccination rates than other racialized children, and vaccine reminders may improve measles vaccine uptake among Black parents. However, there is limited data in Canada on vaccine uptake in children of Black parents, with evidence suggesting vaccine hesitancy among the Black population. See Pg 2 line 26 |
|  | **Reviewer #1**  The introduction emphasizes the importance of vaccines, but the first paragraph seemed to be about the importance of strategies to improve the vaccines themselves which seemed to differ from the purpose of the article? It would perhaps be useful to begin with information about vaccination and then discuss vaccine uptake and whether there are differences among different patient populations. It would also be helpful to provide additional context on the trends in vaccination rates over time | Thank you for the comment. We have addressed the comment on the introduction to include the information below in the introduction.  Vaccination is a critical public health intervention that protects against infectious diseases by stimulating the body’s immune system to develop immunity.[1] According to the World Health Organization(WHO),[2] vaccines prevent diseases by enhancing the body’s natural defense mechanisms. See Pg 4 line 68  However, recent trends indicate a decline in childhood vaccination rates in Canada and the United States of America (USA), raising concerns about potential outbreaks.[10] In the USA, the measles-mumps-rubella (MMR) vaccination rate declined from 79% in 2014 to 73% in 2019 and further to 61% in 2021. See Pg 4 Line 82  During the COVID-19 pandemic, vaccination disparities became evident, with Black children in the USA receiving COVID-19 vaccines at lower rates (1%) compared to Hispanic (2.5%) and White children (4.5%).[11] Similarly, in Canada, data on vaccination coverage among Black children is limited, but available evidence suggests disparities exist. For instance, Statistics Canada reported that Black Canadian children had lower vaccination coverage (73%) compared to other racialized children (86%).[12] See Pg 4 line 85 |
|  | Importantly, (linking to the comment above about the abstract) the general rationale for focusing on vaccine reminders wasn’t clear. There were arguments posed about lack of knowledge about vaccines, and about disparities between parents belonging to different ethnic groups, but the arguments and references provided did not clearly show how providing reminders would resolve these issues. | The statement below on the rationale for parental vaccine reminder has been added to the manuscript to address the consent raised.  Hargono et al.’s study[13] showed that parents with insufficient vaccination knowledge are up to six times more likely to miss immunization opportunities than those with greater awareness. Providing parents with timely vaccine reminders can enhance adherence to immunization schedules and improve overall vaccination rates.[18,19] Pg 5 line 99  However, with a limited understanding of parents’ vaccination reminder interests and preferences, especially the Black parents in Canada and the USA, strategies that are most effective[28,29,30] would remain unexplored. Additionally, there is a lack of evidence on which reminder strategies are most effective in improving childhood vaccination rates in diverse populations.[29,31] See Pg 6 line 119  By synthesizing existing evidence, this review will contribute to developing targeted interventions to enhance childhood vaccination rates, particularly among historically underserved populations. See Pg 6 line 131 |
|  | The focus on North America was also not made clear – why only North America, particularly when some of the other literature cited in the introduction focused on other countries? | Other countries were cited in the introduction to provide background information to the topic. However, Canada and the United States of America were the focus for this review because the authors reside in these countries. Furthermore, there has been recent outbreaks of vaccine preventable diseases in Canada and the USA which necessitates this review to highlight the gap in the literature to guide future research. The statement provided below has been added to the context of the inclusion and exclusion.  Existing literature highlights a lack of research on vaccine reminders for parents in, particularly among the Black population, who may have reservations about vaccination due to historical experience, migration, and socio-ecological factors, including racial discrimination experiences.[29,31]Therefore, the selected North American countries (i.e., Canada and the USA) will be included in the review to identify gaps in knowledge about vaccine reminders for parents, especially Black parents. See Pg 10 line 184 |
|  | The objective referred to examining the effectiveness of vaccine reminder strategies – it might be helpful to include the word ‘effectiveness’ in the title to make it clearer | It is our expectation that the findings from this scoping review will map out the information available on vaccine reminders that work for parents particularly in relation to childhood vaccination. As a scoping review methodology, identifying the effectiveness of strategies or interventions could be one of the objectives but not the main reason for conducting the scoping review. We have revised the objectives to align with main reasons for conducting a scoping review to enable us to maintain the title as it is. See Pg 6 line 124 |
|  | Towards the end of the introduction there is some detail that may be best allocated to the methods section (e.g. beginning on page 6, line 121). | Thank you for the recommendation. The beginning of paragraph 6, line 121 has been moved to the methods section. |
|  | I would suggest moving the ‘significance of the study’ section towards the end of the manuscript (unless this follows a prescribed PLOS One formatting preference that I missed). It also was not clear as to how this study will contribute to understanding of racial disparities in vaccination rates – if this will come out of the results from this study perhaps it will become clearer by moving this section to the end of the protocol paper. The statement about identifying areas for future research I think is making an assumption of what you will find in the results of the scoping review and I would suggest removing it. | The significance of the study has been moved from the introduction towards the end of the protocol. The statement “furthermore, the review will identify areas for future research, including my current PhD project, that focuses on examining the Black Canadian parents’ preferences for childhood vaccination reminder strategies” has been removed from the protocol.  See Pg 15 line 299  The statement below has been added to the significance of understanding racial disparities.  Thus, it is expected that the findings of this study will provide more knowledge about the vaccine reminder strategies that Black parents and other racialized populations prefer to enhance vaccine uptake, particularly for Black parents who often have low childhood vaccinations. See Pg 15 line 302 |
|  | I would also recommend removing the section on ‘identifying the research question’. Figure 1 could be included but might fit better elsewhere. It would also help to specifically align the various RQ’s in Figure 1 with the objectives of this study – some of the RQ listed in figure 1 seemed to extend beyond the plans referred to at the end of the introduction | We concur with the reviewer to move the figure 1.  Figure 1 has been moved to the methods section (see Pg 7, line 148-Fig.1) of the manuscript and the research objectives and questions have been revised to enhance their alignment in the introduction (See Pg 6 line 124). |
|  | **Reviewer #1**  Please provide the rationale for focusing on 2004-2024. An explanation was provided in the methods for why children up to age 6 years were included, but rationale for North American studies only was not provided (which could be in the introduction, as suggested above). | We thank the reviewer for his comments. The review time frame has been revised to the recent 10 years based on the recommendation received from reviewer #3. The statements below has been added to the manuscript.  The ten-year time frame was chosen to ensure the review reflects current practices, preferences, technological trends, and evolving parenting approaches while considering shifts in healthcare access. See Pg 10 line 192 |
|  | The search strategy may find a lot of articles intended for reminders to adults – I wonder if it would be helpful to consider including a term for children and infants? How will the grey literature be searched/used? | Thank you for the suggestion. We will include children and infants in our search terms.  Besides databases, hand-searching of journals and gray literature will be carried out to retrieve pertinent articles and book chapters.  Gray literature will include theses and dissertations from appropriate databases. Manual and electronic searches from public health organizations’ websites such as the Public Health Agency of Canada and the United States Center for Disease Control will be conducted to search for discussion papers, conference papers, commentary, editorials and book reviews. The findings obtained from the grey literature search will improve the quality of information for the review.  Pg 11 line 214  The search terms include child*, infant*, See Pg 10 line 203 |
|  | In general, throughout the methods there seemed to be some confusion over the process for a scoping review vs a systematic review. Both ‘charting the data’ and ‘data extraction’ were referred to – which one will be done? The terminology throughout could be made consistent to fit with the specific study type. Related to this, I was not clear why the authors were using the PRISMA checklist and not PRISMA-ScR if it’s a scoping review – although both appeared in the appendix? | We will use a data extraction table to chart the retrieved data from the articles that will be included in the scoping review. “Charting the data has been deleted”. See Pg 7 line 142  The Prisma checklist has been removed from the revised submission and only the PRISMA-ScR has been added to the submission.  The statement below has been added to the protocol |
|  | **Strengths and limitation**  **Reviewer #1**  The strengths and limitations section is quite brief and could be expanded | The information below has been added to the manuscript.  The study will identify gaps in the literature and highlight racially specific findings by identifying differences in the uptake of immunization reminders between Black parents and non-Black parents. See Pg 16 335  Another anticipated limitation is that the scoping review will be restricted to studies conducted in English due to time constraints.  See Pg 16 line 341 |
|  | **Implications**  **Reviewer #1**  In terms of implications, how do the authors think the findings may inform and improve public health strategies to boost vaccine uptake? | In agreement to the reviewer’s comment, the statement below has been added to the protocol.  Thus, this study will inform public health authorities about the specific preferred reminder strategies for parents, especially Black parents in North America, to develop culturally sensitive vaccination programs as well as to improve childhood vaccinations. For instance, creating awareness to the health authorities about what Black parents prefer e.g., a combination of reminders, such as emails and phone calls, could inform how these strategies are used to involve parents in participating more in childhood vaccinations. In addition, considering that culturally, Black parents tend to emphasize family and community, which aligns with the African philosophy of Ubuntu,[46,47] adding this critical stakeholder key in promoting vaccination is long overdue. Similar findings of reminders from family and social contacts or significant guardians in this review will inform public health authorities to utilize culturally appropriate strategies to advance vaccine uptake among parents, particularly Black parents. See Pg 16 322 |
|  | **Reviewer #2**  The introduction includes some redundant statements, particularly about the benefits of immunization. | Thank you for your comments. We concur with the reviewer that some statements in the introduction are redundant. This statement about the benefits of immunization has been removed from the protocol “Besides, immunization saves the lives of about 3 million children each year worldwide.”  The definition of immunization has also been deleted. |
| . | Reviewer # 2  Using approaches and strategies interchangeably might not be right as the two are different. | The word approaches have been removed and the term strategy has been used in the revision instead of approaches. |
|  | Certain claims, such as the effectiveness of reminders, lack specific evidence or references in the introduction section. | References have been provided in the introduction.  For this study, **effective vaccine reminder strategies** are defined as interventions that successfully improve childhood vaccination adherence.[32] This review will assess these strategies based on parental preferences and findings from intervention studies.[33] By synthesizing existing evidence, this review will contribute to developing targeted interventions to enhance childhood vaccination rates, particularly among historically underserved populations. See Pg 6 Line 131 |
|  | The manuscript does not explicitly mention how findings will be synthesized to inform practical applications or policy recommendations. The discussion lacks depth in addressing the challenges of synthesizing diverse data sources (e.g., qualitative vs. quantitative). | Meta-synthesis will be employed to the interpretation of qualitative data to provide an understanding of parental reminder strategies that improve childhood vaccinations. See Pg 14 line 280  A narrative summary will be used to interpret both the quantitative and qualitative data during analysis. See Pg 14 line 285.  Qualitative data analysis specifically thematic analysis will be employed to interpret and combine both textual and numerical data which were summarized using narrative summary. |
|  | **Reviewer #2**  The implications for Black parents are highlighted but not explored in detail in the discussion. | We concur with the reviewer to provide culturally specific examples. The information below has been added to the protocol.  Thus, this study will inform public health authorities about the specific preferred reminder strategies for parents, especially Black parents in North America, to develop culturally sensitive vaccination programs as well as to improve childhood vaccinations. For instance, creating awareness to the health authorities about what Black parents prefer e.g., a combination of reminders, such as emails and phone calls, could inform how these strategies are used to involve parents in participating more in childhood vaccinations. In addition, considering that culturally, Black parents tend to emphasize family and community, which aligns with the African philosophy of Ubuntu,[46,47] adding this critical stakeholder key in promoting vaccination is long overdue. Similar findings of reminders from family and social contacts or significant guardians in this review will inform public health authorities to utilize culturally appropriate strategies to advance vaccine uptake among parents, particularly Black parents.  See Pg 16 line 322. |
|  |  | We concur with the reviewer to operationalize the ambiguous cases. Under the inclusion and exclusion criteria, the statement below has been added.  However, eligible studies conducted among adolescents and younger children will be included if they report on the younger children as well. Only studies that report findings on younger children will be retrieved and used for this scoping review. See Pg 10 line 194  Grey literature that meet the inclusion and exclusion criteria will be included in the review. Quality assessment of the grey literature will be manually examined using JBI quality assessment tools. |
|  | **Reviewer #3**  The protocol shows promise. However, it suffers from methodological gaps, unclear objectives, and limited justification for some decisions. Addressing these issues comprehensively will significantly improve the manuscript's quality and relevance. | Thank you for the comment. We have included the objective in accordance to the research questions.  This scoping review protocol aims to map the existing literature on vaccine reminder strategies available to parents in Canada and the United States, focusing on racial and socioeconomic disparities. Specifically, the review will: a) Identify the extent of research on vaccine reminder strategies and parental preferences; b) Explore gaps on immunization reminders among parents in the literature, especially concerning Black parents in Canada and the USA.  See Pg 6 line 124  The revised purpose and research questions and objectives have been addressed to enhance consistency. |
|  | Specifically, 1. The inclusion of studies spanning 20 years may no longer be fully relevant given the rapid advancements in technology, particularly in communication and healthcare systems. Over this period, significant changes have occurred, including widespread access to free information and the growing use of digital tools such as smartphones and automated reminders. It is recommended to limit the inclusion period to more recent years to reflect on the current practices, preferences, and technological trends while addressing generational shifts in parenting and access to healthcare resources. | Thank you for the insightful comment. We will limit the time frame for the review to the last ten years 2015-2025. Studies published between 2015 and 2025 will be included. The ten-year time frame was chosen to ensure the review reflects current practices, preferences, technological trends, and evolving parenting approaches while considering shifts in healthcare access. See Pg 10 line 191. |
|  | 2.There appears to be a typographical error under the "Settings" section S1A Table, where the inclusion and exclusion criteria mentioned both Canada and US. | 2.Thanks for the comment It is now written as the  United States of America. See Pg 8 line 163 |
|  | 3. While the title states that the study focuses on "North America," the inclusion criteria are limited to Canada and the United States. The scope should be expanded to genuinely include all North American countries for consistency with the title. | Thank you for the comment.  The title of the protocol has been edited to include Canada and United States of America. See title on Pg 1 line 1  We focused on two (Canada and the United States) of the three North American countries because of language barrier. Mexico was not included because none of the authors spoke or understood Spanish to accurately review articles from this country. |
|  | 4. The title of the manuscript is vague and does not explicitly communicate the central focus on Black parents, despite significant emphasis on this demographic in the text. | 4.The protocol aims to map out literature about vaccination reminders for **all parents**. However, we also aim to obtain information about Black parents given that children of Black parents have lower vaccination rates compared to other racialized population. |
|  | 5. It is unusual and generally unnecessary for a protocol intended for publication to explicitly mention that it is part of graduate work. Publications are expected to focus on the research content itself and its contribution to the field rather than on the context of how or why the study was developed. Please revisit some sections. | 5. The statements about the protocol being part of a graduate work has been deleted. |
|  | **Reviewer #4**  Rationale and Research Questions: The manuscript provides a compelling rationale for the proposed scoping review by emphasizing the public health significance of childhood immunization and the resurgence of vaccine-preventable diseases in North America. The research question is well-structured, clearly identifying the focus on parental immunization reminder strategies. However, greater specificity about the unique contributions of this review compared to existing literature could enhance the manuscript's impact**.** For instance, while the racial disparities in vaccination uptake are discussed, the protocol could expand on how it plans to address the gap in understanding the cultural or systemic barriers specific to Black parents. | Thank you for the comment. Additional information has been provided in the manuscript to address the concern on racial disparities in vaccination uptake and plans to address the gap in understanding the cultural or systemic barriers specific to Black parents.  For example, in the significance section of the study we added the statement below:  Thus, it is expected that the findings of this study will provide more knowledge about the vaccine reminder strategies that Black parents and other racialized populations prefer to enhance vaccine uptake, particularly for Black parents who often have low childhood vaccinations. See Pg 15 line 301  Specific recommendations and lay summaries will be made to inform nursing practice, education, research and administration about the preferred reminder strategies for parents, most especially Black parents, for childhood vaccinations, including Black community agencies, respectively. In addition, the findings of the study will be disseminated at relevant conferences using oral and poster presentations and the manuscript will be published in a peer-reviewed journal. See Pg 15 line 307  Furthermore, we have indicated that the findings from the study will inform future research on the gap in the literature on immunization reminders. |
|  | **Reviewer #4**  Methodological Framework: The manuscript demonstrates a rigorous approach, adopting the JBI methodology and building upon the framework by Arksey and O’Malley. However, it would benefit from further details about how the inclusion and exclusion criteria will be operationalized in ambiguous cases (e.g., studies addressing both adolescents and younger children). | The comment has been addressed in reviewer # 2 comments. See Pg 10 line 194 |
|  | Additionally, the authors might consider discussing the potential challenges of using gray literature and how they will ensure its quality. | The comment has been addressed in the statement below:  Additionally, grey literature that meets the inclusion and exclusion criteria will be included. These will comprise of theses and dissertations, conference papers and editorials, including published data, will be included to ensure that the data for the review is reliable and extensive. Commentaries will be excluded as they may not provide reliable data. Pg 7 line 154  Grey literature that meets the inclusion and exclusion criteria will be included in the review. Quality assessment of the grey literature will be manually examined using JBI quality assessment tools. |
|  | **Reviewer #4**  Significance and Implications: The study's significance in addressing racial disparities is important, but the manuscript could better articulate how its findings might be operationalized into practical interventions or policies. For example, it could outline how public health authorities could use these insights to design culturally sensitive vaccination programs | Addressed under reviewer number 2 comments. See Pg 16 line 322. |
|  | **Reviewer #4**  Data Availability and Reproducibility: While the protocol mentions using a systematic approach for data extraction and review, it does not explicitly state where the data underlying the review's findings will be made publicly available after completion. Including this information would align with best practices in transparency and reproducibility. | Thank you for the comment. The authors have provided the information below under a new heading availability and reproducibility.  The authors will keep the data that underlies the review in safety in a password protected computer. Data from the review will be shared with the public using the University of Regina data sharing space, Dataverse "All data for this protocol are in the manuscript and/or supporting information files"  See Pg 17 344 |
|  | Parental Reminder Strategy Definitions: The manuscript defines reminders broadly as digital and non-digital strategies. While this inclusivity is commendable, providing examples or a taxonomy of reminder strategies in the methodology section would help clarify how the study will categorize diverse strategies encountered in the literature. | We concur with the reviewer to include examples of digital and non-digital reminders in the methods. The examples have been provided in the information below under the concept of the inclusion and exclusion criteria in the methods section.  Vaccine reminders encompass digital (e.g., text messages, mobile apps) and non-digital (e.g., mailed notices) strategies that inform parents of upcoming immunization appointments.[20]  See Pg 5 line 102 |
|  | Writing and Presentation: Overall, the manuscript is well-written and intelligible. However, certain sections could benefit from tighter editing to improve clarity. For instance, the repeated references to studies with similar findings might be synthesized for conciseness. Additionally, using consistent terminology throughout the manuscript (e.g., interchangeably using "reminder strategies" and "reminder preferences") would reduce potential confusion. | Thank you for the comment. We have taken note of the comment and will be consistent in the use of reminder strategies and reminder preferences. We would like to explain that the two terms are different, and we tried to use each term appropriately in the revised version of the manuscript. Reminder strategies refer to the digital and non-digital modes of reminding parents. For example, text messages are used as reminders strategies. Besides, the authors refer to reminder preferences as reminder strategies preferred by the parents. For instance, a parent might prefer text message whereas another parent might prefer telephone calls. |
|  | Engagement of Black Parents: Given the focus on racial disparities, the authors should consider elaborating on how they will account for potential biases in the included studies that could affect the representation of Black parents. Explicitly stating a plan to address or discuss these biases in the analysis would strengthen the study's conclusions. | We are grateful for the comment and drawing our attention to the potential of introducing bias in the selection of data to be included in the review. The review is focused on all parents and not just Black parents in North America. Thus, the first step is to include all articles that meet our inclusion criteria. It is the product of the review that we will comment on the literature we identified on Black population. The authors intend to include studies involving all parents. The overarching objective indicates that  This scoping review protocol aims to map the existing literature on vaccine reminder strategies available to parents in Canada and the United States, focusing on racial and socioeconomic disparities. Specifically, the review will: a) Identify the extent of research on vaccine reminder strategies and parental preferences; b) Explore gaps on immunization reminders among parents in the literature, especially concerning Black parents in Canada and the USA.  See Pg 6 line 119 |
|  | Figures and Tables: While the manuscript includes references to figures and tables, their clarity and relevance are not fully detailed in the narrative. It would be beneficial to ensure all supplementary materials are cohesive and directly support the primary text. | The comment has been addressed. For example, see Pg 14 line 283-289  The table will summarize information about authorship, publication information, study purpose, methods (population, concept, context), findings or key arguments and conclusion.  This protocol will provide a consistent method for the data extraction process to address the question of what childhood immunization rem**i**nders are available to parents in Canada and the USA. Pg13 line 245  Data such as type of reminders, immunization, age of children, and vaccine uptake will be extracted from both published and unpublished studies. Pg 13 line 262 |
|  | Recommendations: Clearly state where and how the final dataset will be made accessible. | Thank you for the recommendations.  As indicated above, the data will be made available to the public using the University of Regina Data sharing space with the public, Dataverse. Pg 17 line 344 |
|  | Elaborate on potential cultural or systemic barriers affecting Black parents' engagement with immunization reminder systems. | The recommendations about cultural and systemic barriers have been addressed.  We have included in the manuscript such as in the introduction literature on barriers affecting Black parents’ engagement with immunization in Canada and USA. Another critical issue influencing vaccine uptake is racial and socioeconomic disparities. Research indicates that Black parents in Canada and the USA are more likely to experience barriers to vaccination access, contributing to lower immunization rates among their children.[21] Data from the Government of Canada revealed that Black children had lower coverage for key vaccines, such as rotavirus (69%) and Hib (65%), compared to White children (86% and 77%, respectively).[22] In the USA, vaccine coverage among Black children was reported at 70.54%, compared to 82.74% among White children.[23] Structural barriers, such as limited healthcare access, lower income levels, and vaccine hesitancy, further exacerbate these disparities.[24] Pg 5 line 105 |
|  | Provide a more detailed breakdown of the types of reminder strategies and how they will be categorized during analysis. | We have provided examples of reminder strategies we will be extracting from the data. Comment has been addressed above. See pg 5 line 102 |
|  | Address how quality will be assessed for gray literature and other non-peer-reviewed sources. | Information about assessing the quality of gray literature is provided.  Gray literature will include theses and dissertations from appropriate databases. Manual and electronic searches from public health organizations’ websites such as the Public Health Agency of Canada and the United States Center for Disease Control will be conducted to search for discussion papers, conference papers, commentary, editorials and book reviews. The grey literature that will be included are those that have been published to improve the quality of information for the review. Pg 11 line 215 |
|  | Ensure consistency in terminology and provide clearer links between the research questions, methodology, and anticipated outcomes. | Consistency of terminologies have been provided. The research question and objectives have been clearly linked. Pg 6 line 127 and Figure 1 on Pg 7 line 148 |
|  | Section-Specific Areas of improvement: Rationale and Research Questions The introduction does not clearly specify the unique contribution of this review to the existing body of literature. While it mentions disparities among Black parents in North America, it lacks detail on the specific knowledge gaps this study aims to address. | Thank you for the comment. It has been addressed in the introduction section under the reviewer #1 comments. See Pg 6 line 131  This review will assess these strategies based on parental preferences and findings from intervention studies.[33] By synthesizing existing evidence, this review will contribute to developing targeted interventions to enhance childhood vaccination rates, particularly among historically underserved populations. |
|  | Methodological Framework The inclusion and exclusion criteria are broadly stated without providing detailed handling of ambiguous cases, such as studies involving mixed age groups or interventions not exclusively targeting parents. | The comment to handle ambiguous cases has been addressed under reviewer #2 comments. See Pg 9 line 170 |
|  | Data Availability The manuscript mentions data extraction and analysis but does not address plans for data sharing or transparency in making extracted data available for reproducibility. | The comment on data availability has been addressed in a reviewer’s comment. See Pg 17 line 344 |
|  | Parental Reminder Strategy Definitions The types of reminder strategies included are vaguely described as "digital and non-digital methods" without offering concrete examples or a taxonomy to guide the reader. | The comment has been addressed See Pg 10 line 194  Also, the examples have been provided in the information below under the concept of the inclusion and exclusion criteria in the methods section.  This study defines reminders as digital and non-digital communication strategies used to notify parents about vaccination appointments. Digital reminders include text messages, telephone calls and email notifications, while non-digital reminders include appointment endorsements in health cards and family-based reminders.  See Pg 9 line 176. |
|  | Significance and Implications The discussion of implications is generic, stating that findings will address disparities in vaccination uptake, but it does not specify how these findings might inform practical or policy-level interventions. | The comment has been addressed in the statement below.  Thus, it is expected that the findings of this study will provide more knowledge about the vaccine reminder strategies that Black parents and other racialized populations prefer to enhance vaccine uptake, particularly for Black parents who often have low childhood vaccinations… Specific recommendations will be made to nursing practice, education, research and administration about the preferred reminder strategies for parents, most especially Black parents, for childhood vaccinations. In addition, the study's findings will be disseminated at relevant conferences using oral and poster presentations and the manuscript will be published in a peer-reviewed journal. More importantly, the gap in the literature on parental vaccine reminder preference in North America will be identified and informed future research.  See Pg 15 line 302-314 |
|  | Writing and Presentation The manuscript contains repetitive references to the effectiveness of digital reminders without synthesizing or streamlining these findings to improve readability and flow. | The comment has been addressed be deleting repetitive statement improving the flow of readability. |
|  | Engagement of Black Parents The manuscript does not evaluate or discuss potential biases in the included studies concerning the representation of Black parents or how these biases might impact findings. | The recommendation has been addressed above in the reviewer #4 comments. |
|  | Figures and Tables The figure summarizing the search strategy lacks sufficient detail to allow for an independent replication of the methodology, such as the specific databases searched and the keywords used. | The recommendation has been addressed above in reviewer #4 comments. Pg 14 line 283-289. In addition, the statement below has been added to the manuscript.  The protocol involves a preliminary search using the keywords in the search strategy on one database. Page 11 line 207. |
|  | **Reviewer #5**  Authors have taken only 20 years data. Is it possible to take 50 years of data? Still the article looks good for publication | Thank you for your comment. More detailed explanation has been provided on the implications of the findings. We have chosen to go for the last 10 years based on feedback from reviewer #1 to ensure the review reflects current practices, preferences, and technological trends while addressing generational shifts in parenting and access to healthcare resources. See Pg 11 Line 215 |
|  | Additional requirements  Please ensure that your manuscript meets PLOS ONE's style requirements, including those for file naming. The PLOS ONE style templates | Thank you for the comment. Manuscript has been formatted to align with PLOS ONE’s format. |
